# Prevalence of Occupational Injuries of the Oral and Maxillofacial Region and Their Covariates Among Building Construction Workers in Chennai

**DOI:** 10.7759/cureus.49468

**Published:** 2023-11-27

**Authors:** P Rahmath Meeral, Srisakthi Doraikannan, Meignana Arumugham Indiran

**Affiliations:** 1 Public Health Dentistry, Saveetha Dental College and Hospitals, Saveetha Institute of Medical and Technical Sciences, Saveetha University, Chennai, IND

**Keywords:** sustainable buildings, migrant workers, secure work, safe work, social protection systems, work related injuries, occupational hazards, dental injuries, oral maxillofacial injuries, construction workers

## Abstract

Background and aim

With many risky environmental conditions, civil construction sites are prone to physical injuries, especially those pertaining to the oral and maxillofacial regions. The current study was an effort to assess the magnitude and pattern of such oral and maxillofacial injuries and the factors associated with them.

Methodology

This descriptive study was carried out on 524 construction workers, of whom 254 met the inclusion criteria related to work site injuries. An interviewer-administered proforma with basic demographic details is used in conjunction with an intraoral examination to classify the dental injury. Descriptive statistics were done to evaluate the frequency of injury occurrence, while inferential statistics, including the chi-square test and regression analysis, were done to evaluate the association between injury and the variable under concern.

Result

The study includes a total of 254 participants, with ages ranging from 20 to above 50 years, of whom 230 (91%) were males and 24 (9.4%) were females. The majority, 200 (78.7%), were unskilled laborers, and 195 (76.7%) were migrant workers with language barriers. It was found that 95 (76.7%) had a history of dental injury alone, while 59 (23.2%) had a history of oral maxillofacial injury. Among the reasons for injury, the increased odds ratios (OD) were noted in the collapse of the surrounding area as 0.050 (0.029-0.075), rainy season 1.001 (0.891-1.281), unskilled labor 1.020 (0.910-1.30), and migrants 1.010 (0.901-1.200). The OD for males is 2.052 (1.941-2.101).

Conclusion

The current study confirms that the magnitude of workplace-related injuries is significant, and the majority of them stem from basic language barriers among migrant workers and a lack of knowledge to adhere to safety protocols and instructions given.

## Introduction

The never-ending surge for rural to urban migration, and spatial urban settlement expansion, ensures at least 6 million urban residents in the year 2041 [1]. The construction industry with its giant migrant workforce will act as a major contributor to this shift. Infrastructure growing at a never-before-seen scale in the country and with a population rate that has tripled since 1950 is itself proof of rapid change [2].

The Indian state of Tamil Nadu is headquartered in Chennai. The city and its suburbs, which make up the urban agglomeration, are home to almost 8.9 million people, making it the fourth most populated metropolitan region in the nation and the 31st largest urban area in the world, according to the provisional results of the 2011 Census [3]. Any industry associated with infrastructural growth and national development comes with a huge labor force and workplace-related issues. According to the International Labor Organization, construction workers are three to four times more likely than other workers to have fatal injuries or casualties in workplace accidents, making construction employment hazardous in most contexts. The risk increases by three to six times with questionable safety equipment, negligible knowledge, and adherence to precautions, especially in developing and underdeveloped countries [4].

Also, workers who suffer trauma from work-related dangers are more likely to become disabled, with over 270 million injuries resulting in a loss of three and a half years of healthy life for every 1000 workers, and construction workers are at high risk of this concern [5,6]. The Building and Other Construction Workers (Regulation of Employment and Working Conditions) Act, 1996, was introduced in Parliament as a response to a high incidence of workplace injuries and health and safety issues among construction workers [7].

Despite the strict regulations and laws pertaining to safety precautions to be followed on construction sites, the per-annual incidence of injuries and casualties holds a worrisome picture and also poses a significant economic and public health problem linked to a person's quality of life and productivity at various levels [8]. This injury incidence also most of the time involves dental structures resulting in dental injuries, which is evident from the previous study by Ugolini et al. in 2018 [9]. These dental traumas are often underreported in comparison to other injuries in various body areas that necessitate emergency care.

A comprehensive baseline analysis pertaining to workplace injuries and labor safety and adherence to standards related to this would shed light on the issue and ensure a holistic measure is devised in the form of policy or regulation. Hence, the current study was planned among major construction sites in Chennai (South), with a primary objective to assess the prevalence and a secondary objective to assess the association between the injuries and other potential covariates like age, gender, migrant status, nature of work, and barriers to adherence to safety standards.

## Materials and methods

This is a cross-sectional study conducted among the construction workers of various building organizations in the southern part of Chennai city. The study was conducted during the event “Celebration of Builders” in Chennai during the month of October 2022, where the majority of the construction workers gathered. The ethical clearance for conducting the study was obtained from the Scientific Review Board prior to the initiation of the study with the trial number SRB/SDC/PHD-2102/22/042.

**Figure 1:**
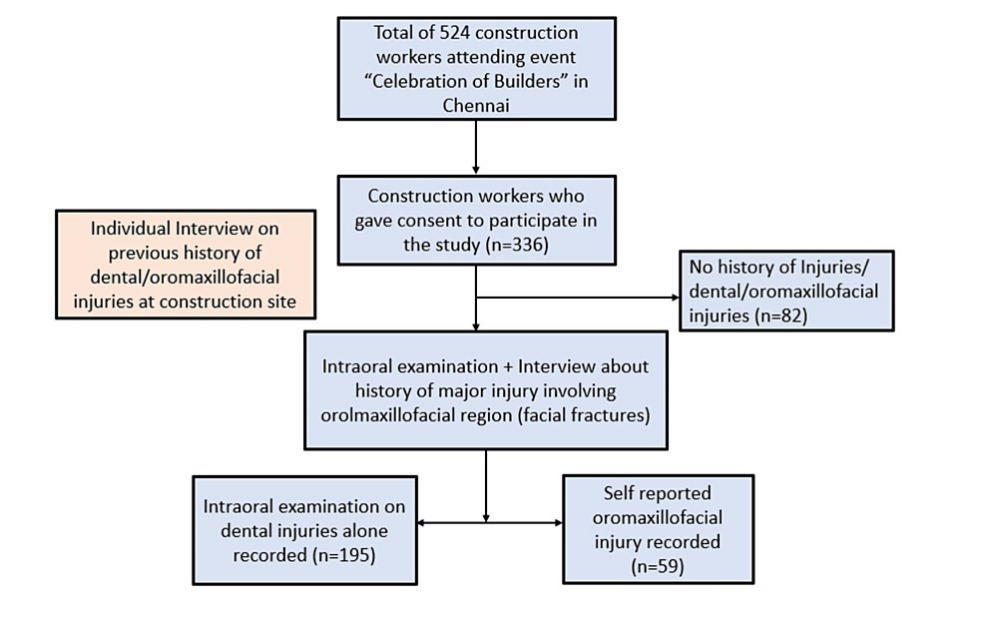
Sample recruitment of the study

Permission and support for conducting the study were obtained from the organizing association. All the construction workers were briefed about the conduct of the study, and informed consent was obtained before the initiation of the study. Convenience sampling was used, where all the construction workers attending the event were interviewed individually about the history of injury in the oral maxillofacial region, and only those who had injury experience were included in the study. Of the 524 workers screened, 254 met the inclusion criteria, and they were included in the study. The sample recruitment is shown in Figure 1.

Information regarding their age (20-30 years, 31-40 years, 41-50 years, >50 years), gender (male or female), geographical origin (migrants from other parts of Tamilnadu or native of Tamilnadu), the season of occurrence of injury (rainy: July to October, summer: March to June, winter: November to February), working category of construction workers (mason, unskilled labor, bricklayer), and reason for injury (fall slip due to wet area, fall slip due to lack of barricade/gaps in staircase/slipping of ladder, accidental fall of any building material, accidental hit by vehicle or moving machine, lifting of a heavy object, the break of rope/harness, collapse of the surrounding area, equipment mishandling, electric shock) were investigated and recorded. Following the interview, a subsequent intra-oral examination was conducted by two calibrated examiners, achieving acceptable inter-examiner reliability with a kappa value of 0.87. The examination aimed to note the type of dental injury and any self-reported maxillofacial injury. The types of dental injuries were categorized according to the epidemiological dental injury classification provided by the World Health Organization (WHO) [10].

All data were statistically analyzed using SPSS Statistics version 23.0 (IBM Corp. Released 2015. IBM SPSS Statistics for Windows, Version 23.0. Armonk, NY: IBM Corp.). The test for normal distribution was done using the Kolmogorov-Smirnov test. Descriptive statistics and inferential statistics using the chi-square test were used. Additionally, regression analysis was done by a multinominal logistic regression test to find the unadjusted odds ratio between the categorical values of various independent variables (age, gender, work category, origin of workers, season of injury, reason for injury) and dental and maxillofacial injury at construction sites. A p-value of <0.05 was considered statistically significant.

## Results

The current study included a total of 254 subjects, of which 19 (7%) belong to the age group 20-30 years, 74 (29%) belong to 31-40 years, 78 (30.7%) belong to 41-50 years, and 82 (31.8%) belong to >50 years. Among them, 230 (90.5%) were males, and 24 (9.4%) were females. Among them, 195 (76.7%) were migrants, and 59 (23.2%) were non-migrants. Among them, 29 (11.4%) belong to the mason category, 200 (78.7%) belong to unskilled laborers, and 25 (9.8%) belong to the bricklayer category. All the categories of variables showed a statistically significant difference of p-value of 0.000 in having an injury (both dental and oral maxillofacial injury) event at the construction site. Table 1 shows the frequency of various categories in different variables assessed.

**Table 1:** Distribution of various factors associated with injuries at construction sites and its strength of association A non-parametric chi-square test was used to evaluate the statistical association between the various categories of different variables. A p-value of ≥0.05 is considered as statistically significant. * shows the significant values. An unadjusted odds ratio was found to evaluate the strength of association within the various categories of different variables on having injury event (both dental and oral maxillofacial injury considered together). The odds ratio signifies the number of odds of having an injury when compared to the reference.

Variable	N=254 (%)		Total n (%)	p-value	Odds ratio (95 % CI)
	Dental injury alone n (%)	Oral maxillofacial injury with or without dental injury n (%)			
Age					
20 to 30 years (reference)	17 (7)	2 (0.78)	19 (7)	0.000*	1.023 (0.930-1.202)
31 to 40 years	61 (24)	13 (5)	74 (29)		
41 to 50 years	59 (23)	20 (8)	79 (30.7)		
Above 50 years	58 (23)	24 (9.4)	82 (31.8)		
Gender					
Male	175 (69)	55 (22)	230 (90.5)	0.000*	2.052 (1.941-2.101)
Female (reference)	20 (8)	4 (1.5)	24 (9.4)		
Season					
Rainy: Jul to Oct	112 (44)	28 (11)	140 (55.1)	0.000*	1.001 (0.891-1.281)
Summer: Mar to Jun	53 (21)	23 (9)	76 (29.9)		0.801 (0.691-1.001)
Winter: Nov to Feb (reference)	30 (12)	8 (3.1)	38 (14.9)		
Working category					
Mason	23 (9)	6 (2)	29 (11.4)	0.000*	- 0.010 (-0.009-0.007)
Unskilled labor	157 (62)	43 (17)	200 (78.7)		1.020 (0.910-1.30)
Bricklayer (reference)	15 (6)	10 (4)	25 (9.8)		-
Origin					
Migrants	149 (58.6)	46 (18.1)	195 (76.7)	0.000*	1.010 (0.901-1.290)
Native to Chennai (reference)	46 (18)	13 (5)	59 (23.2)		
Reason for injury					
Fall slip due to wet area	10 (3.9)	(0)	10 (3.9)	0.000*	- 0.009 (0.007-0.010)
Fall slip due to lack of barricade/gaps in staircase/slipping of ladder	20 (7.8)	23 (9)	43 (16.9)		-
Accidental fall of any building material	4 (1.5)	18 (7)	22 (7.8)		-0.110 (0.04-0.117)
Accidental hit by a vehicle or moving machine	18 (7)	1(0.3)	19 (7)		-0.0107 (0.010-0.011)
Lifting of a heavy object	7 (2.7)	(0)	7 (2.7)		-0.001 (0.0006-0.008)
Break of rope/harness	5 (1.9)	15 (5.9)	20 (6.6)		-0.0106 (0.0102-0.113)
Collapse of the surrounding area	73 (28.7)	2 (0.7)	75 (29.5)		0.050 (0.029-0.075)
Equipment mishandling	15 (5.9)	(0)	15 (5.9)		-0.0101 (0.0100-0.011)
Electric shock (reference)	43 (16.9)	(0)	43 (16.9)		

**Figure 2:**
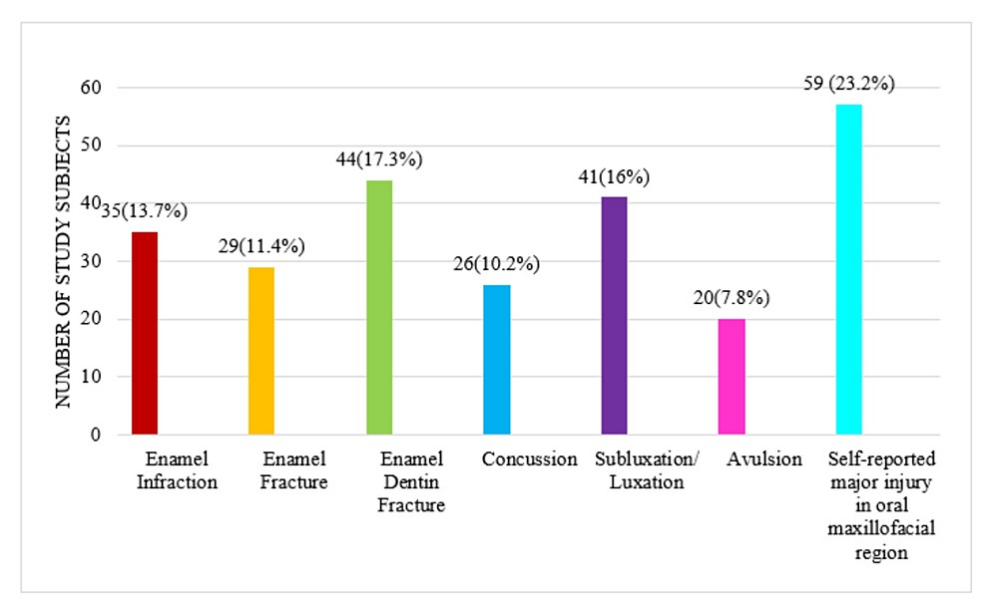
Frequency distribution of various types of injuries at the construction site A total of 195 (76.7%) had dental injuries alone while 59 (23.3%) had experienced major injuries involving the oral maxillofacial region (facial fractures).

Among them, 195 (76.7%) had a history of dental injury alone, while 59 (23.2%) had a history of oral maxillofacial injury during their construction working hours. The frequency of various types of injuries is demonstrated in Figure 2. It was found that enamel infraction in 35 (13.7%), enamel fracture in 29 (11.45%), enamel dentin fracture in 44 (17.3%), concussion in 26 (10.2%), subluxation or luxation in 41 (16.1%), avulsion in 20 (7.8%), and self-reported major injury in the oral maxillofacial region in 59 (23.2%) were noted.

Among the total construction workers, 140 (55.1%) injuries occurred during the rainy (July to October) season, 76 (29.9%) during the summer (March to June), and 38 (14.9%) during the winter (November to February) season, where a statistically significant difference occurs between the seasons with a p-value of 0.000. With the winter season as a reference, the rainy season and summer season have odds of having an injury of 1.001 (CI 0.891-1.281) and 0.801 (CI 0.691-1.001), respectively.

Compared to females, males have higher odds of experiencing injuries, with an odds ratio of 2.052 (CI 1.941-2.101). With an increase in age by one year, the odds of having a dental or oral maxillofacial injury increase by 1.023 times (CI 0.930-1.220).

**Figure 3:**
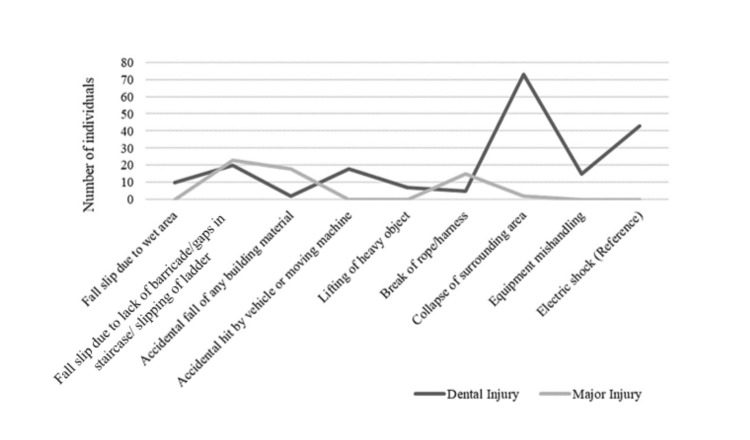
Various reasons for injuries at the construction site Collapse of the surrounding area was noted as a major reason for dental injury, while fall slip due to lack of barricade/gaps in staircase/slipping of ladder forms a major reason for major oral maxillofacial injury.

Regarding the reason for the injury, fall slip in a wet area, accidental hit by a vehicle or moving machine, lifting of a heavy object, equipment mishandling, and electric shock resulted only in dental injuries with a frequency of 10 (3.9%), 19 (7%), seven (2.7%), 15 (5.9%), and 43 (16.9%), respectively. Fall slips due to a lack of barricades, gaps in the staircase, or slipping of the ladder resulted in 20 (7.8%) dental injuries and 23 (9%) oral maxillofacial injuries. An accidental fall of any building material resulted in four (1.5%) dental injuries and 18 (7%) oral maxillofacial injuries. A break in the rope or harness caused five (1.9%) dental injuries and 15 (5.9%) oral maxillofacial injuries. The collapse of the surrounding area resulted in 73 (28.7%) dental injuries and two (0.7%) oral maxillofacial injuries. There exists a statistical difference with a p-value of 0.000 between the reasons for injury. Figure 3 shows the various reasons for injuries at the construction site.

Regression analysis showed, with the reason "electric shock" as a reference, that there exists no association with the reason “fall slip due to lack of barricade/gaps in staircase/slipping of the ladder,” a negative association with “fall slip due to the wet area,” “accidental fall of any building material,” “accidental hit by a vehicle or moving machine,” “lifting of a heavy object,” “break of rope or harness," and "equipment mishandling” with odds ratios of -0.009 (CI 0.007-0.010), -0.110 (CI 0.04-0.117), -0.0107 (CI 0.010-0.011), -0.001 (CI 0.0006-0.008), -0.0106 (CI 0.010-0.113), -0.010 (CI 0.01-0.011), respectively. However, with the reason “electric shock” as a reference, “collapse of the surrounding area” had a positive association that the latter has 0.050 odds of causing injury (odds ratio 0.050, CI 0.029-0.075). Table 1 shows the distribution of various factors associated with injuries and their strength of association. Figure 2 shows the distribution of various reasons contributing to injuries at construction sites.

## Discussion

The fundamental purpose of this national policy on safety, health, and environment at the workplace is not only to eliminate the incidence of work-related injuries, diseases, fatalities, disasters, and the loss of national assets but also to ensure the achievement of a high level of occupational safety, health, and environment.

It is crucial to address the occupational hazards linked to the building industry because they contribute significantly to the expansion of the global economy and workforce employment. Working in complex places, working at heights, physical handling, lifting, groundwork, demolition work, and other tasks are all part of the construction process. All these activities have dangers and are extremely dangerous. Therefore, there is a significant likelihood of accidents occurring on building sites. To avoid repeating the same mistakes, a detailed inquiry that pinpoints the causes must be conducted. To prevent the incidence of work site injuries in the civil construction field, there have been many protocols to be followed, as recommended by various policies. However, it was not followed strictly in many instances, which led to the incidence of work-related injuries.

A systematic review by Bravo et al. found age to be a non-contributing factor for both fatal and nonfatal injuries, as most of the included studies in their review showed no association between age and injury at construction sites. However, this review also explains the probable association between increasing age and decreasing working efficiency that might influence the rate of injury with age [11].

The result of the current study shows that most of the injuries occur in the rainy season, which is in line with the previous retrospective analysis in Delhi by Edwards et al. in 2022, which shows that most of the injuries at the construction site occurred in males during the rainy season, were non-fatal injuries, and were due to the collapse of the surrounding area [12]. Similarly, a 2019 study by Ammar et al. found that the number of accidents was highest in the spring, followed by the summer, and lowest in the winter [13]. However, a study by Tian et al. in 2023 showed that there was a greater risk of injury during construction work with increasing weather precipitation (during winter), which is contradictory to the results of the current study [14].

According to studies, using outdated and unsafe equipment and failing to maintain construction equipment both increase the risk of injuries on the job site [15]. Getnet et al.'s systematic review in 2020 concluded that inadequate safety orientation, training, and supervision play a significant role in injury occurrence [16]. These findings give a plausible explanation for the greater number of injuries that have occurred due to various reasons, like fall slip due to a lack of barricades or gaps in the staircase or slipping of the ladder, fall slip due to a wet area, accidental fall of any building material, accidental hit by a vehicle or moving machine, lifting of heavy objects, break of rope or harness, equipment mishandling, electric shock, and collapse of the surrounding area.

According to a 2009 study by Chi et al., fatalities were caused by risky behaviors such as failing to deactivate electrical equipment, failing to maintain safe distances, using PPE incorrectly, and using subpar work practices [17]. Another study by Promsorn et al. in 2015 concluded that inadequate site management was one of the causes of construction work-related accidents [18].

A study by Senoucia et al. in 2015 found that poor environmental management, which includes a lack of barricades, gaps in staircases or slipping of a ladder, vehicle hits, moving objects, building collapses, and electric shocks, is increasingly associated with construction site injuries [19].

Previous studies have also found that the lack of personal protective equipment has an association with injury at construction sites, which can protect against injuries from various etiological factors that were assessed in the current study [20,21]. A study by Jain et al. in 2014 also gave the same result, as construction work was commonly associated with fall slip from height [22].

The current study also showed that migrant workers had an increased injury incidence compared to non-migrants. This was in line with the previous study by Kim et al. in 2020, in which they found that, when compared with regular workers, migrant workers have a higher risk of occupational injury and mortality [23]. This might be due to the poor understanding of the safety measures given because of the language barrier [24]. According to Cheng et al.'s 2013 research, inadequate safety and health education has made migrant workers more susceptible to accidents [25]. Also, they often fail to complain about unsafe working conditions [26]. Saleh et al. in 2001 found increased rates of occupational injury occurrence between the ages of 36 and 45 [27]. Abbas et al. in 2013 found that falls from height and mishandling of equipment account for 47.6% and 23.8% of injuries at construction sites, respectively [28].

The limitation of the current study is that there might exist a bias in the self-reported information provided by the study subjects regarding their history of maxillofacial injury. A future study will be designed with a thorough investigation of the medical records to be conducted to assess the risk of maxillofacial trauma occurring at a construction site.

## Conclusions

Within the limitations of the current study, it can be concluded that the construction site is more prone to various types of injuries, with oral maxillofacial injuries occupying a considerable ratio. Thus, the result of this current study recommends having proper safety protocol instructions provided to various construction workers in their language during the civil upliftment process. Also, there should be strict regulations on wearing personal protective equipment, which can protect against many risk factors that can cause injuries.
